# Application of Nanohydroxyapatite in Medicine—A Narrative Review

**DOI:** 10.3390/molecules29235628

**Published:** 2024-11-28

**Authors:** Adam Lubojański, Wojciech Zakrzewski, Kinga Samól, Martyna Bieszczad-Czaja, Mateusz Świtała, Rafał Wiglusz, Adam Watras, Bartosz Mielan, Maciej Dobrzyński

**Affiliations:** 1 Department of Pediatric Dentistry and Preclinical Dentistry, Wroclaw Medical University, Krakowska 26, 50-425 Wrocław, Poland; maciej.dobrzynski@umw.edu.pl; 2 Pre-Clinical Research Centre, Wroclaw Medical University, Bujwida 44, 50-345 Wroclaw, Polandksamol58@gmail.com (K.S.); mbieszczad8@gmail.com (M.B.-C.); m.switala97@gmail.com (M.Ś.); b.mielan@umw.edu.pl (B.M.); 3 Institute of Low Temperature and Structure Research, Polish Academy of Sciences, Okólna 2, 50-422 Wrocław, Poland; r.wiglusz@intibs.pl; 4 Department of Organic Chemistry, Bioorganic Chemistry and Biotechnology, Faculty of Chemistry, Silesian University of Technology, Krzywoustego 4, 44-100 Gliwice, Poland; 5 Meinig School of Biomedical Engineering, College of Engineering, Cornell University, Ithaca, NY 14853-1801, USA

**Keywords:** nanohydroxyapatite, nanotechnology, dentistry, bone regeneration, implants

## Abstract

This review is an extensive collection of the latest literature describing the current knowledge about nanohydroxyapatite in a comprehensive way. These are hydroxyapatite particles with a size below 100 nm. Due to their size, the surface area to mass ratio of the particles increases. They are widely used in medicine due to their high potential in regenerative medicine, as a carrier of various substances, e.g., in targeted therapy. The aim of this article is to present the biological and physicochemical properties as well as the use of nanohydroxyapatite in modern medicine. Due to the potential of nanohydroxyapatite in medicine, further research is needed.

## 1. Introduction

Biomaterials are becoming more and more popular in medicine, due to their wide range of utilization and their properties can be introduced into human tissues. Hydroxyapatites, thanks to their bioactivity, biocompatibility, stability, and non-toxicity is particularly noteworthy. Hydroxyapatites are inorganic compounds which are the main component of hard tissue as tooth enamel and bones, as well as dentine. Bones are composed of 30% organic (which are proteins—mainly collagen, polysaccharides, and lipids) and 70% inorganic substance—such as apatite (approx. 65% bone mass and their content varies depending on the type of bone, as well as age, diet and physical activity of the person) [1]. Hydroxyapatites have many applications. They are used as bone substitute materials (due to their biocompatibility), sunblinders, anti-wrinkles. It is also used as a toothpaste ingredient to treat tooth hypersensitivity. It was found that nanohydroxyapatite could determine a progressive closure of the tubular openings of the dentin and the regeneration of a mineralized layer. It also may be a carrier of drugs, e.g., chemotherapeutics in targeted cancer therapy. Hydroxyapatites also support the healing of chronic and difficult to heal wounds, forming a scaffold for soft tissues when closing the wound. More and more often it is also used in aesthetic procedures in the form of a colloidal suspension, which forms a scaffold after injection and stimulates the formation of collagen [2].

Hydroxyapatite (HAp) is an inorganic calcium apatite mineral naturally occurring in human hard tissues, like teeth or bones. It has a structural formula Ca_10_(PO_4_)_6_(OH)_2_. Almost half of its composition39.92%, is calcium, and 18.45% is phosphorus [3]. Because of its bioactive and osteoconductive properties and similarity to the inorganic component of human bones in vivo, it is one of the most common apatites used as a bioceramic in medicine and dentistry. The hydroxyapatite crystals store practically all calcium found in the organism and constitute more than 70% of the bone-building material. As a material, it is characterized by biocompatibility, bioactivity, and bioconductivity. It consists of small crystallites of ion-substituted HAp, which is created by replacement of a fraction of carbonate groups with either phosphates or hydroxyl groups. It is worth mentioning that not only is nanohydroxyapatite a material with great potential in medicine, but e.g., tricalcium phosphate or bioglass are also characterized by good biocompatibility and osteoconduction. Chemical similarity is one of the advantages of HAp over other bioceramics, like bioglass. Natural hydroxyapatite can be obtained, for instance from fish bone, chicken bone, or eggshells [4,5,6,7].

Nanotechnology has shown a promising future, when it comes to improving materials used for bone regeneration. Nanomaterials have unique characteristics when compared to micro-sized materials due to particle sizes of 1–100 nm. The nanoparticles used in HAp are low crystalline with highly active surfaces. Among many nanoparticles with different material composition, inorganic nanoparticles that are composed of calcium phosphate are characterized by several advantages like biocompatibility, ease of synthesis, and the possibility of controlling their physicochemical properties. Nanoparticles have many possibilities for biological application. Techniques based on these particles show huge promise in either biomedical or bioassay application [8]. Nanohydroxyapatite can be enriched with various dopants that have beneficial effects on properties such as antimicrobial, osteogenic differentiation, and osteoblast response. Nanoparticles that can be added to nanohydroxyapatite include Cu^2+^, Zn^2+^, Eu^3+^, Sr^2+^, Zn^2+^, and Si^4+^. This demonstrates the great potential of nanohydroxyapatite and its great potential in medical applications [9,10,11,12].

**Figure 1:**
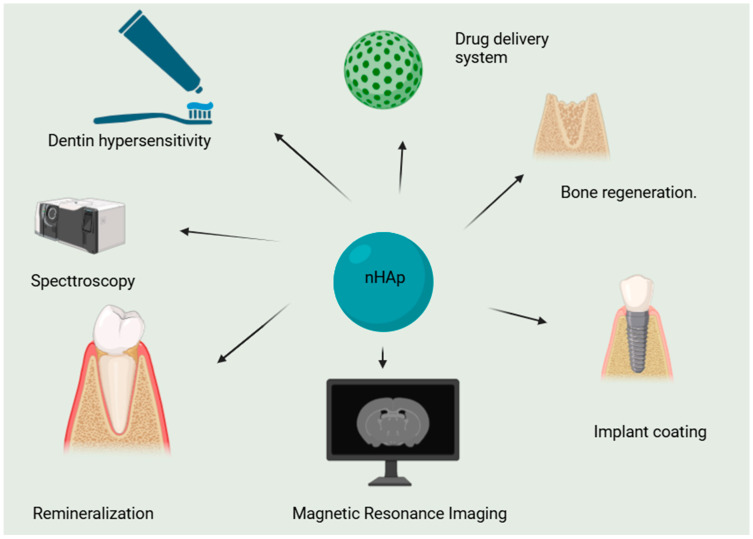
Examples of medical applications of nanohydroxyapatite in dentistry.

Nanohydroxyapatite(n-HAp) is widely used as scaffold for bone tissue engineering. The quality of the scaffold mostly depends on certain characteristics, like crystallinity, particle morphology, and particle-size distribution or density [13]. Among the methods of synthetic nano-crystalline HAp, the most commonly used are mechanochemical and various techniques of wet chemistry [14], such as hydrothermal, hydrolysis [15,16], or aqueous colloidal precipitation. Figure 1 shows examples of the utilization of nanohydroxyapatite in various branches of dentistry, demonstrating its versatility and potential for various applications.

**Figure 2:**
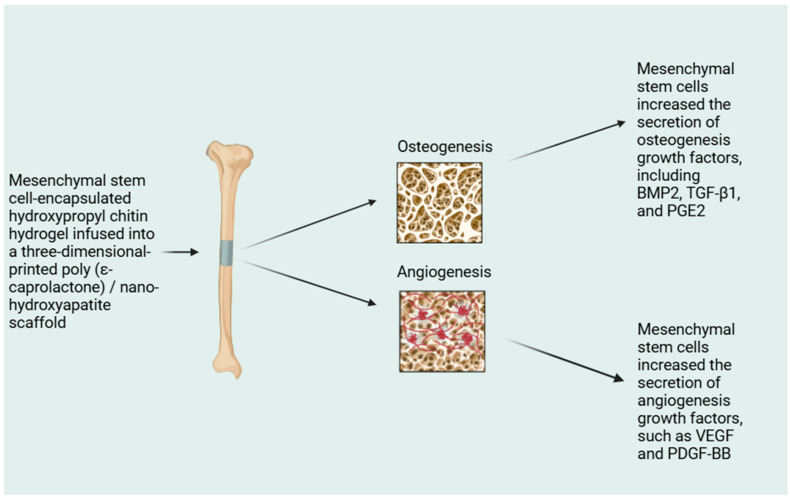
A scheme presenting the utilization of modern materials in bone regeneration.

**Figure 3:**
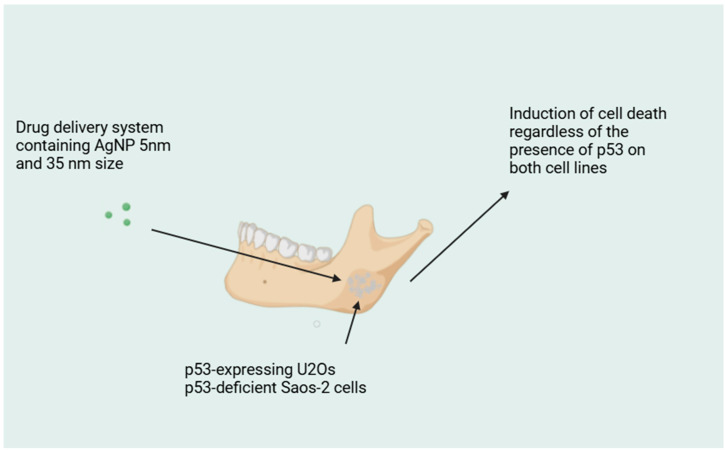
Shows a scheme for drug delivery system containing AgNP.

**Figure 4:**
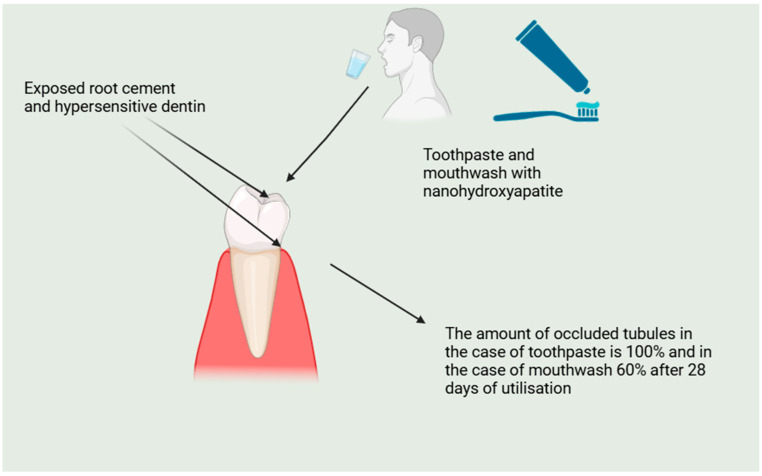
Demonstrates the application of toothpaste and mouthwash with nanohydroxyapatite and comparison of its effectiveness.

**Figure 5:**
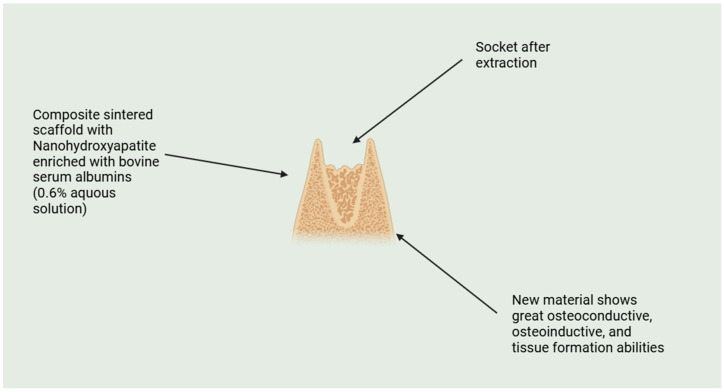
Shows socket after extraction and application of modern composite sintered scaffold with added nanohydroxyapatite and growth bovine serum albumins.

This review aims to bring together current knowledge of nanohydroxyapatite and to describe selected applications in medicine. The data for this narrative review were collected by many specialists in various fields of science. EBSCO, PubMed, and current medical books databases were used for this purpose. The website Biorender.com was used in the creation of Figure 1, Figure 2, Figure 3, Figure 4 and Figure 5.

## 2. Properties of Nanohydroxyapatite

### 2.1. General Properties

Both synthetic and mineralogical apatite crystallizes in the hexagonal system, allowing for easy substitution of its differentiated cations. The cations that can be substituted are Na^+^, Mg^2+^, K^+^, and Sr^2+^, while the anions are CO_3_^2−^, HPO_4_^2−^, Cl^−^, and F^−^.

The unit cell of HAp (synthetic or natural origin) most commonly has a hexagonal crystal structure, with space group P63/m and lattice constants a and c equal to 0.942 and 0.688 nm, respectively. The structure consists of arrays of PO_4_ tetrahedra held together by Ca ions interspersed among them. These ions are placed in two different sites, in aligned columns, in axes and the adjacent OHs point in opposite directions [17]. HAp has two types of crystal planes, positive charges on a and b planes, while there are negative charges on c planes. Positive charges attract the molecules with negative charge, for instance acidic molecules, while negative charges attract particles with positive charges, for instance basic molecules [18].

The hexagonal crystal structure of HAp is highly flexible, stable, and it allows for a wide variety of substitutions that will alter its physical properties and bioactivity. The substitutions can involve divalent or trivalent cations for the Ca^2+^ ions and anions for either hydroxyl or phosphate groups. One of the examples can be the substitution of fluorine into the hydroxyl sites, leading to improved crystallinity, or substitution of strontium into the Ca sites that causes increase of bioactivity [19]. In a single-cell unit, there are 14 calcium ions. Eight of them are peripheral and shared among adjacent unit cells. The other six ions are placed inside the unit cell. Out of all 10 PO_4_^3−^ groups of a unit cell, 8 are peripheral and 2 of them are located inside. The OH^−1^ groups remain at the edge of unit cell, shared by four unit cells each. Each unit cell of HAp consists of 10 calcium ions, 6 phosphate ions and 2 hydroxyl ions [20]. Pure hydroxyapatite is white and slightly soluble in water, it is practically insoluble in alkaline solutions, but it dissolves very well in acids. It is characterized by the sorption of fatty acids, saliva, and lipids which may be important for their use in dental implants. In the human body environment, it is practically insoluble and does not release calcium, which makes it an ideal material for use on permanent implants. Hydroxyapatite is capable of forming chemical bonds with surrounding hard tissues [21]. Stoichiometric apatites crystallize in a monoclinic system; they have Ca/P ratio of 1.67, which is the most stable at normal temperatures and pH between 4 and 12 [22]. Although the ratio differs in human bone tissue, usually reaching between 1.5 and 1.67 [23].

Monoclinic apatite is primarily formed by heating the hexagonal form at 850 °C in air, and then cooling to room temperature. This type of apatite can be a rarity, because a slight deviation from the stoichiometry results in the formation of a hexagonal structure. Hexagonal HAp is usually formed by precipitation from supersaturated solutions at 25–100 °C [24]. The size, morphology, chemical composition, and crystal formation of the HAp greatly influence its characteristics and possibility of application. For instance, nanoscale HAp crystals have extraordinary biocompatibility and sintering ability, even better than HAp of microscale size particles [25].

In medical applications, HAp is characterized by extraordinary osteoconductive and biocompatibility. Studies have shown that nanohydroxyapatite also has osteoconductive properties [26]. Although it has many major strengths, it is not a perfect biomedical material. Among its disadvantages, the following can be distinguished: low degradation rate and low mechanical properties. Although traditional synthetic HAp can be widely used as a bone graft, due to its bioactivity and high octeoconductivity, but it still problematic to induce stem cell differentiation within large bone defects [27,28]. Figure 2 shows the potential of mesenchymal stem cell-loaded thermosensitive hydroxypropyl chitin hydrogel combined with a three-dimensional-printed poly(ε-caprolactone)/nanohydroxyapatite scaffold in bone regeneration. The authors report that in addition to greater endurance, angiogenesis and osteogenesis are more intense [26].

Osteoinduction requires the ability to concentrate several bone growth factors under physiological conditions. Furthermore, these factors lead to accumulation of mesenchymal stem cells (MSCs) and osteoblasts, which increases proliferation and expression of osteogenic genes. Biological apatites are substituted by essential trace elements on trace levels. Improvement in bone regeneration can be achieved by tailoring the surface structure of the grafts. HAp with nanoscale crystal sizes usually promotes better differentiation of both osteoblasts and bone marrow stem cells, which eventually leads to better bone regeneration [29]. In order to increase the bioactivity of HAp, it can be doped with either growth factors, endothelial cells, or mesenchymal stem cells [30]. Another method that allows simple improvement in the bioactivity of HAp is electrical polarization. Polarized HAp has extraordinary effects on bone-like crystal growth in stimulated body fluid. Growth is accelerated on the negatively charged surface of the HAp and decelerated on the positively charged surface. Finally, it was confirmed that manipulation of cells on the material surface can be possible with use of polarization effect.

Resorption is a difficult process when it comes to HAp. It impedes its use in applications like tissue engineering scaffolds or drug carriers. Using a second phase with a faster degradation rate, like calcium carbonate or sulfate, is a quick method of regulating the degradation rate of HAp-based materials. The HAp degradation rate is dependent on several factors, including HAp form, purity, and the microstructure of body conditions [31]. Change in grain size diameter and ion substitution are two of the methods of HAp degradation control. Nano-sized particles possess higher dissolution rates when compared to micro-sized HAp particles. The solubility rate is influenced by a number of boundaries between the particles of a given material. In a nano-sized material, there are more particle boundaries than in a micro-sized particle [32]. HAps with co-substituted essential trace elements of natural bone, like Na, Mg, K, or F, can be successfully synthesized via a facile hydrothermal process at low temperature, in the absence of any surfactant or organic solvent. This biomimetic HAp porous microspheres can be very suitable for applications as catalysts or biomaterials [33].

When it comes to HAp biomaterial, it is usually applied in the form of either porous scaffolds or granules. Macroporous scaffolds are especially hazardous as load-bearing materials because of their mechanical properties. Usually, such a HAp requires application of reinforcement in the form of Al_2_O_3_ or TiO_2_ [34]. Nano-sized bioceramics exhibit higher mechanical properties in case of stresses when compared to micro- or macro-sized HAp. The mechanical strength of pure HAp bioceramics obtained by various technologies is still lower than that of natural bones. Although n-Hap hardness is lower along the crystallographic direction, the fracture toughness appears to be higher [35]. HAp ceramics are often coated as a film on metal materials to increase the biocompatibility and osteoconductivity of the dental implants [36].

### 2.2. Properties of Size and Shape

Particle behavior in blood flow is affected by their size. Particles > 500 nm in diameter are marginated towards the walls by gravitational force, whereas for particles < 500 nm, the Brownian motion causes localization towards the flow chamber wall. The removal of drug delivery systems from the body is also affected by the size of particles. Nanoparticles < 10 nm can be removed by renal clearance, but those bigger than 200 nm are mainly accumulated in the spleen or engulfed by phagocytic cells. The optimal size range for particles is considered to be 10–100 nm, as they are able to penetrate through very small capillaries, have longer circulation time, and are not accumulated in the body system [37,38]. The shape of nanoparticles plays an important role in their behavior in body. Spherical nanoparticles have better hydrodynamic flow than particles shaped like rods, plates, flowers, or wires, etc. On the other hand, spherical particles show a greater phagocytic activity than macrophages and a shorter blood circulation time compared with rod-shaped particles [39,40,41]. Pore size seems to be an important characteristic of nanomaterials in drug delivery. Mesoporous inorganic materials, with pore size 2–50 nm, due to their high pore volume and adequate pore size, can store a higher number of therapeutic molecules. Moreover, mesoporous particles can be easily functionalized with different ligands, which allows us to attach drug molecules [20,42]. Hollow HAp nanoparticles, such as hollow spheres and nanotubes, were demonstrated to have superior drug-holding efficiency than solid nanoparticles in similar sizes [43]. Hollow nanoparticles can be obtained by using a template-directed approach. Templates are coated by HA and then selectively removed. This results in the creation of hollow particles with a large fraction of voids in their inner space, which can encapsulate more therapeutic agents [44,45].

### 2.3. Surface Property

The surface charge of nanoparticles is an important property to consider when creating a drug delivery system. It can affect the stability of particles in colloidal solutions, their distribution in the body system, the capability of therapeutic agents’ attachment, and the internalization of nanoparticles in target-specific cells. The most common measure of surface charge is the surface electrokinetic potential (zeta potential). It is calculated and quantified on the basis of electrophoretic mobility [46]. It has been shown that nanoparticles that are positively charged can be easier taken up by cancer cells and the reticuloendothelial system. On the other hand, strongly positive nanoparticles are usually toxic to cell membrane. In contrast, slightly positive particles do not show this toxicity. HAps have been measured to have a zeta potential of around −21.5 mV. This can be reversed to be positive after surface functionalization [47]. A much less common technique is surface titration, where the buffering capacity of a surface is determined by its ability to soak up the H^+^ (and/or OH^−^) from a solution at a range of pHs. Harding, I. S. et al. used this technique and concluded that although the point of zero charge (PZC) for HAp is pH = 7.3 ± 0.1, it accumulates positive charge more readily below the PZC than it accumulates negative charge above it [48]. Surface chemical modification or ionic substitutions can change the zeta potential of the HAp surface. It can facilitate drug absorption or change other properties of the drug delivery system. Moreover, the high positive or negative zeta potential of nanoparticles do not agglomerate in storage, because of high dispersion stability. The hydrophobic surface of nanoparticles makes them easier to be opsonized, followed by clearance through macrophage engulfment. This results in a low circulation time in the cellular environment. On the other hand, hydrophilic nanoparticles are able to escape engulfment. Therefore, they show longer retention time in the body system which results in better therapeutic efficiency [20].

### 2.4. Biodegradation and Cytotoxicity

Biocompatibility is an important factor in a drug delivery system, as nano-scale particles can be internalized by the cells. HAp is well-studied biocompatible material. In the body system, HAp degrades with the release of Ca^2+^ and PO_4_^3−^ ions, which favor bone cell formation and do not show any toxic effect [49,50,51]. Commercially available HAs have been tested for mutagenicity and carcinogenicity and they did not show these side effects [52]. Studies also did not show any inflammatory effects of Hap [53]. Moreover, HAp was studied for its anticancer inhibitory effect. Once internalized by the cell, the HAp will degrade slowly, increasing Ca^2+^ concentration. The elevated concentration of intracellular Ca^2+^ ions can be the factor to stimulate apoptotic signals. It can synergize with anticancer drugs carried by Hap [54]. It shows that HAps can be safely used as drug delivery systems.

## 3. Application of Nanohydroxyapatite

### 3.1. Spectroscopic Techniques Used for n-HAp Characterization

Spectroscopy is a branch of science which inquires and measures the spectra produced by materials which interact or emit electromagnetic radiation. One of the first methods in this field was Raman spectroscopy, invented in 1928, which is still developing because of its low cost and high efficiency [55,56]. Spectroscopy, in combination with microscopy and new components, for example the interferometer and array detector-equipped microscope, can increase the possibilities of developing new technologies utilizing materials such as hydroxyapatite [57]. There are many types of spectroscopy; for example, Fourier-transform infrared spectroscopy (FT-IR), which can help distinguish ions in biomaterials, bone tissue regeneration, and synthetic apatite products. The Attenuated Total Reflection (ATR) mode in FT-IR is very useful in studying new materials such as carbonate-substituted hydroxyapatite. Due to spectroscopy, it can be confirmed that the carbonate substitution covers the whole surface of the composites which is observed in the wavenumber of 1500–1400 cm^−1^ [58]. Vibrational spectroscopy (Raman and IR) allows us to recognize the functional groups in a material. The structure and spectroscopic properties can change when silicate-substituted hydroxyapatite has a different amount of dopants, for example Eu^3+^. This method can prove that ions have been incorporated into the structure, and also indicate the exact place [59,60]. Another type is Terahertz time-domain spectroscopy (THz-TDS) which characterizes low cost and high accuracy; for example, it can help in defining the amount of residual HA in biomaterials during their resorption without calcinations [61,62]. Theranostic drugs are vibrantly developing nowadays, and spectroscopy helps us to examine new methods. HA, as one of the calcium phosphate bioceramics, are suitable for carrying drugs. The UV-VIS spectroscopy can be used to estimate the concentration of drug [63]. Laser ablation–inductively coupled plasma–mass spectrometry (LA-ICP-MS) is great tool for monitoring trace elements in for example tooth. This method ensures the correlation of metal distribution in tissues which is very useful in the study of the impact of the environmental exposure on the tooth [64]. LA-ICP-MS is versatile method which allows imaging and quantifying elements from biomaterials like HA. It is fast and needs little amounts of preparation [65]. Spectroscopy is a versatile tool, and it is very useful in the study of hydroxyapatite nanoparticles; it can be exploited depending on the purpose and properties of the material. HAp:Ln-AMP-poly (IA-MPC) composites are characterized by luminescence, which can be researched by fluorescence spectroscopy [66,67,68]. Depending on the needs, spectroscopy provides wide access to instruments which can help to develop biomaterials such as HA nanoparticles.

### 3.2. Carbonate A and B

Synthetic bone grafts are currently made from Ca_10_(PO_4_)_6_(OH)_2_. The most common method of synthetic hydroxyapatite fabrication is ions incorporation into the HAp structure. Carbonate ions are naturally present in bone material [69]. CO_3_ can substitute the phosphate group or hydroxide group. The first type is about substitution of carbonate ions for phosphate ions, called B-type. The process usually takes place at elevated pH [70,71]. If a carbonate ion is substituting for a phosphate ion, there has to be a reduction in the number of calcium ions to maintain charge balance. It can be compensated when sodium ions are used from sodium carbonate, which was used in the precipitation as a source of carbonate ions [72].

Ca_10−x_Nax(PO_4_)_6−x_(CO_3_)_x_(OH) [73].

The second type, called A-type, is focused on substitution of carbonate ions for hydroxyl groups. OH groups are exchanged for carbonate groups in stechiometric HAp. This method requires a special environment—a CO_2_ atmosphere with temperature of 900–1000 °C for 15–144 h [73]. Another method can be soaking pure sintered HAp powder in an aqeous solution, which was saturated in carbon dioxide for up to 2 months. This is a simple procedure leading to the formation of material with controlled chemical composition.

Both the A and B types of synthesis can occur simultaneously, causing the formation of a mixed AB complex [73,74,75]. HAp and Carbon-substituted hydroxyapatite(CHAp) differ in sintered density from 3.03 to 3.16 g/cm^3^. CHA sinters to high densities at temperatures around 200 °C, which is lower than what is required for pure HA. Comparison of grain size revealed that when CHAp was sintered at 1000 °C, it had smaller grain size than when the HA sample was sintered at 1200 °C.

A comparison of bioactivity measurement was conducted, comparing HAp and CHAp to determine the time required to form a poorly crystalline apatite layer on the surface of the material while it is immersed in a simulated body fluid (SBF). Gibson et al. [73] proved the visual appearance of needle-like apatite crystals on CHAp surface within 7 days, while it took from 24 to 28 days for pure Hap to appear. The pH did not change much over the 28-day period for both materials. SBF solution after testing showed, that in both samples there was initial minimal increase in calcium and phosphorus ions as time immersion in SBF increased. Later however, it was followed by rapid reduction in these ions below the original phosphorus and calcium levels, which occurred faster in the CHAp sample—around 7 days, than in the pure HAp—around 17–21 days.

The simple synthesis route of the CHAp indicates that it is an improved synthetic alternative to pure HAp, and can work more efficiently as a bone graft implant.

### 3.3. Nanohydroxyapatite Doped with Copper

One of the primary features of HAp is its capacity for ion substitution, usually divided into total and partial exchange. The latter one is limited due to differences in the sizes of the ions, spatial structures, and their charges [76]. HAp has a high affinity for heavy metals like copper.

Copper, similarly to zinc, is a heavy metal ion, one of the inorganic antimicrobial materials [77]. In small amounts, copper is an essential microelement, needed in important metabolic processes; however, in larger quantities, it can be potentially toxic. Copper antimicrobial activity mechanism works either by interaction with bacterial membrane, deactivation of microbial proteins, or eventually, due to interfering with nucleic acids—microbial replication stops. Its inhibitory action is correlated with the amount of H_2_O_2_ production. The bacterial survival rates on the surface of the coating are almost zero when the amount of H_2_O_2_ exceeds 10^−6^ mmol/cm^2^ [78]. Among the affected organisms were both Gram-negative bacteria, like Klebsiella pneumoniae, Pseudomonas Aeruginosa or *E. coli*, and Gram-positive bacteria, including methicillin-resistant Staphylococcus aureus.

The antimicrobial effect of copper is also visible, when its incorporated into drinking glasses, which prevents the appearance of Streptococcus sanguis biofilm formation, which can directly cause oral infections. Mulligan et al. [79] proves that there was a strong correlation between the copper content of the glasses and the viable counts of bacteria in the biofilm. Although there was an increase in the number of bacterial cells after the initial reduction, their level never reached the amount of bacteria found either on pure hydroxyapatite or non-copper-containing glass discs. Copper is highly effective against *S. sanguis*, but there are theories proposing that bacteria may be able to withstand higher level of ions [80,81,82].

### 3.4. Nanohydroxyapatite Doped with Other Rare Earth Metals

There are fourteen elements in the Lanthanides group, from cerium to lutetium (Ce-Lu, Z = 58–71). These elements are useful in a variety of biomedical approaches, e.g., anticancer treatment [83,84]. Lanthanide ions, Ln(III), possess abilities similar to Ca ions, which allow them to influence the bone remodeling process. The HAp structure allows for a wide numbers of substitutions, either in the cationic sublattice of the calcium position or anionic sublattice in the hydroxyl or phosphate groups. Because the lanthanum complex La^3+^ ([tris(1,10-phenanthroline)lanthanum(III)]trithiocyanate (KP772) can be absorbed by tumor cells without a problem and stop a cell cycle, it can be used as a potential anticancer drug [85]; incorporation into HAp enhances its characteristics like its tensile and bending strength [86] biological, physicochemical properties, and stabilized apatite structure [87]. Due to the chemical structure and porosity, HAp can accept many ionic substitutes, e.g., La^3+^. Lanthanium; despite belonging to the lanthanide group, it does not exhibit luminescence because of a lack of unpaired *f* electrons. Zhang et al. [86] proved that La^3+^ concentration of 1.00 × 10^−5^ mol/L can considerably lower bone resorption, which eventually makes La-doped HAp a very promising material in osteoporotic cases. Generally, the doping of lanthanide significantly improves its chemical and thermal stability [88]. Weiwei Leu et al. [86] proved that the La-HAp coatings can increase osteogenic differentiation and proliferation when the content is below 20%.

The photoluminescence in the visible and near-infrared regions is a well-known property of lanthanide ions. Eu^3+^ and Tb^3+^ ions are the most intense emitting elements [89].

Doping HA with Tb resulted in developing photoluminescent properties. The material was homogeneous with rod- and sphere-like morphologies. Tb doping had no important influence on the structure of hydroxyapatite. The excitation with visible light (488 nm) stimulated the green emission of HA nanoparticles. Moreover, these Tb-HA particles could be incorporated by living cells and still show luminescent properties [90]. Similar properties can be developed by doping HA with erbium. Mesoporous-, spherical-, and rod-like nanoparticles were obtained by a microwave-assisted wet precipitation and co-precipitation approach. Material showed near-infrared luminescence emission [91,92]. The studies showed that the luminescent, Eu-doped HA presents a very similar drug loading amount and cumulative release rate as the pure HA. Moreover, the ibuprofen-loaded samples still showed red luminescence under UV irradiation [93]. Lanthanum is another promising metal for doping HA. It promotes osteoblast proliferation. La-doped HA showed increases in crystallinity, crystallite size, and specific surface area. I was concluded that the material is promising in cellular internalization and as a drug-releasing agent [94].

Sr:HA can promote osteoblast cell proliferation and stem cell differentiation. This can enable new bone formation [95]. Moreover, it can also prevent bone resorption by reducing the osteoclast activity [96]. The wide range of Sr concentration rate in HA did not show cytotoxicity [97].

In addition to these advantages, Sr doping also improves the mechanical properties of HA. These effects potentially offer help in the treatment of osteoporosis and osteosarcoma [98]. The synthesis of a mesoporous, Sr-doped HA drug release system with self-activated luminescence was reported by C. Zhang, C. Li, and S. Huang et al. This material, with a nanorod shape, shows a strong blue emission peaking at about 432 nm under UV excitation. It was synthesized via a hydrothermal route with the presence of trisodium citrate. This system can be used in monitored and tracked drug delivery [99].

### 3.5. Magnetic Hydroxyapatite

HA nanoparticles can obtain magnetic properties after doping with specific metal ions. The most common metal for this purpose is iron. Other magnetic dopants investigated in this field are platinum (Pt), manganese (Mn), cobalt (Co), gadolinium (Gd), neodymium (Nd), samarium (Sm), copper (Cu), and their combinations with iron/iron oxides. Iron oxides have superior characteristics over other magnetic compounds, such as less toxicity, high magnetic saturation, and easy fabrication. Magnetic nanoparticles can be used in numerous therapeutic applications: targeted drug delivery, detoxification of biological fluids, magnetic resonance imaging (MRI), cancer therapy, and magnetic fluid hyperthermia. Before their biological application, they require surface modification, such as coating to improve their biocompatibility and reduce toxicity [100]. Magnetic HA was investigated as a drug delivery system. It showed a pH-sensitive drug and protein release property, which can help in transporting drugs to tumor or cancer sites. These nanocarriers showed drug release kinetics similar to other HA-based systems, with an early burst release followed by a slow release. Hollow magnetic hydroxyapatite micro-spheres with hierarchically mesoporous microstructure for pH- responsive drug delivery [101]. Excess or unused drugs could be easily recovered by an external magnetic field. Xu et al. synthesized magnetic functionalized HA nanoparticles for magnetic resonance diagnosis of the acute hepatic injury. The incorporation of iron oxide in the HA structure resulted in the enhanced performance of MRI. This newly synthesized material can be used as a potential contrast agent for MRI [102]. An interesting use for magnetic HA is magnetic hyperthermia to treat cancers. Study showed a temperature increase of 40 °C in 60 s when Fe^2+^/Fe^3+^-doped HA was exposed to an alternating magnetic field [103]. Hou et al. reported the in vivo effectiveness of iron-doped HA in hyperthermia therapy treating cancer in a mouse model. The used therapy resulted in a reduction in tumor volume, and magnetic HA showed good biocompatibility when subcutaneously injected [104]. Magnetic HA nanoparticles can be used to improve the DNA loading and transfection effectiveness for gene delivery. The stable DNA-protecting property of magnetic HA was reported by Zuo et al. It was concluded that this material is a promising magnetic-guided vector for virus-free gene delivery [105]. Table 1 shows possible applications of nanohydroxyapatite doped with different metal nanoparticles.

## 4. Drug Delivery System in Chemotherapy

### 4.1. Drug Loading Capacity

Drug loading capacity is a property of drug system delivery nanoparticles, which measures the amount of the therapeutic agent bound to 1 g of delivery particles. It is dependent on particle features and is different for various drugs. It was proven that hollow HA particles have a greater loading capacity than solid particles [44]. Loading capacity is influenced by the concentration of the therapeutic agent in the initial solution, pH value, buffered solutions, and ionic strength. It was shown, using the example of a model drug, doxycycline hydrochloride (Dox-Hcl), that adsorption of Dox-Hcl increased with an increase in the initial concentration of Dox-Hcl. When the initial concentration rose above critical level, part of the drug precipitated out from the solutions as crystalline particles instead of being adsorbed into HAp. The study also showed that the amount of absorbed Dox-Hcl is greater according to increasing pH from 5.8 to 8.0 [108].

The drug-loading efficacy can be enhanced by doping HA crystals with other metals ions. For example, doping with selenite ions (Se^4+^) showed an increase in lysozyme uptake [109].

Doping Mg^2+^ ions into HA increases the surface positive charge and hence increases its DNA loading capacity [110]. Through changing the chemical structure of HAp and adjusting the pH and the ionic strength of the initial drug solution, it is possible to tailor the amount of the drug loaded into HAp.

### 4.2. Mechanism of Drug Attachment to Hydroxyapatite

There are four main mechanisms for attaching therapeutic agents onto an inorganic surface.

− Ligand-like binding—interaction between ligand and binding part is the result of hydrophobicity, charge, and molecular structure. Bindings involved in this type of interaction are ionic bonds, hydrogen bonds, and Van der Waals forces.
− Electrostatic adsorption—stronger than covalent bonding, it occurs between positively charged biomolecules and negatively charged nanoparticles, or vice versa.
− Covalent binding—adding functional groups to biomolecules and nanoparticles results in a covalent bond, which is the sharing of electron pairs between atoms.
− Noncovalent, affinity-based receptor-ligand systems—this bond does not involve electron sharing, which is important to maintain the three-dimensional shape of proteins and nucleic acids [111].

Covalent bonds seem to be the most beneficial, as they provide the controlled release of proteins or drugs [112]. This linking strategy involves bonding ligands directly with functional groups (amino or hydroxyl groups) present on functionalized surface of HAp. This approach involves increasing loading capacity, protecting the drug’s functionality, and increasing its efficiency.

Physical interactions can also lead to the coupling of drug molecules with the surfaces of nanoparticles. These include electrostatic, hydrophobic/hydrophilic, and affinity interactions. Affinity interactions are the most stable noncovalent linkages, unaffected by environmental conditions, such as the pH and ionic strength of the medium [20]. The encapsulation of drugs into HAp can be approached in two ways. One method is encapsulation of the drug into previously synthesized HA nanoparticles, which is preferred in fabrication of hollow nanoparticles. Another method involves encapsulation of drug during the synthesis of HA nanoparticles. Both methods were proven to successfully encapsulate drugs by HA nanocarriers [45].

### 4.3. Mechanism of Drug Release

Drug delivery systems are expected to be stable at physiological pH = 7.4. The therapeutic agent is released from the HA nanocarrier when the pH trigger point is reached. pH-dependent drug release is an important addition to cancer treatment. In cancer cells, the pH is lower than in normal tissues due to the high rate of glycolysis, both in aerobic and anaerobic conditions [113].

HA particles are relatively insoluble at physiological pH but can be dissolved as nontoxic in acidic conditions [114]. The degradation of carrier particles results in the release of attached therapeutic molecules.

Another approach is to functionalize HAp with chemical groups that form covalent, acid–labile bonds with drug molecules. The most commonly used acid–labile linkers are acetal, orthoester, hydrazone, imine, and cis-aconyl bonds [115]. The drug-release profile of HA nanocarriers systems shows two phases of release. Initially, part of the drug is rapidly released (initial burst release), followed by sustained release. The initial burst must be related to the fraction of drug molecules adsorbed on the surface of HAp. The slower sustained release concerns the drug entrapped in the pores of the HA particles’ structure [54,116].

The presented characteristic of therapeutic molecules’ release is a big advantage of the hydroxyapatite-based drug carrier system. It favors the therapeutic effect for an extended period of time and allows for cell-specific anticancer therapy. It also reduces the systemic side effects of anticancer drugs.

### 4.4. Surface Modification

Functionalization means attaching one or more organic components to the surface of HAp. The addition of specific molecules develops new features for hydroxyapatite-based nanocarriers. The surface of HA is hydrophilic, so to retain liposoluble drugs it needs to be coated with hydrophobic molecules. A commonly used substation for this purpose is polyethylene glycol (PEG). PEG is nontoxic, nonimmunogenic, and nonantigenic. Coating nanoparticles with PEG reduces their agglomeration and clearance by macrophages. It increases the zeta potential of the particles. An additional advantage of this operation is that it increases the circulation time because of resistance to plasma protein deposition. It also allows HA nanoparticles to carry lipophilic therapeutic agents like the anticancer drug docetaxel.

Coating HAp with PEG is a very simple process. Mixing HAp with the 1% concentration PEG 6000 solution in the mass ratio 1:1 and stirring it overnight, followed by washing it with distilled water and freeze drying, allows us to manufacture PGE-coated HAp [117]. PEG-functionalized HAp shows an elevation in the amount of gentamicin loaded into the nanocarrier and the enhanced release duration of the drug [118]. Functionalization with positively or negatively charged amino acids can lead to changes in the surface charge of HAp. The presence of additional carboxyl (COO^−^) and amino (NH_3_^+^) groups of the amino acids on the surface provides binding sites for covalent and electrostatic bounds with transported drugs. Coating nanoparticles with glucuronic acid aids in excreting them through urine, which reduces their toxicity [119,120]. HAps functionalized with folic acid are capable of recognizing cancer cells. Cancer cells show overexpression of the folate receptor (FR). FR is negligible expressed in healthy cells. This makes folic acid a perfect ligand for targeting cancer cells with nanoparticle drug-carrying systems [117]. HAp functionalized with amine (3-aminopropyl-triethoxysilane) was used to create a drug and gene delivery system for candesartan and p53 plasmid. That way, modified HAp showed higher loading capacity and greater protective effect for p35 plasmid [47].

### 4.5. Chemotherapy Cancer Treatment

Chemotherapy in cancer treatment causes many side effects because of its toxicity. The main problem is nephrotoxicity and neurotoxicity; also, too-low drug release induces difficulties. In recent years, studies proved that hydroxyapatite (HA) nanoparticles, because of their structure and properties, can increase the efficiency of drug delivery. Hydroxyapatite is biocompatibile, does not demonstrate toxicity, and is inert in the body fluid environment [45]. The efficiency of HA in chemotherapy depends on its structure. Most of the nano-carriers have a spherical construction which is different from viruses and bacteria occurring in nature. The rod and filamentous shape can avoid immunogenic response, it can also increase the drug capacity, lengthen the in vivo half-life, and toughen the targeting capability. Due to the porosity of HA, it has better drug adsorption, high surface area, and greater capacity [67,68]. Studies confirmed that the high osteoinduction and osteoconductivity of HA nanoparticles allows them to be a drug delivery system for targeted cancer treatment in bones. Bisposphonates (BP) are molecules with the characteristic configuration of phosphorous–carbon–phosphorous and the possibility to bind the hydroxyapatite in bones. Figure 3 demonstrates a drug delivery system containing silver nanoparticles (AgNP) of size 5 and 35 nm in n p53-proficient U2Os and in p53-deficient Saos-2 cells [121].

They are widely used to treat bone disorders like osteoporosis, Paget’s disease, and bone metastases. Nanoparticles can deliver to the target and protect BP from enzymes. Hydroxyapatite-based biodegradable methoxy poly(ethylene glycol)-b-poly(lactide-co-glycolide) (mPEG-PLGA) nanoparticles of risedronate have high bioavailability and a significant ability to keep in osteoporosis [122,123], due to low solubility in the biological system and its favored longer degradation time. These properties make HA an excellent carrier through diffusion and it can be delivered to the target in a large dose. The dual delivery system is a promising branch, where the combined operation of drugs and proteins in simultaneous release enhance its activity in osteogenesis. A ceramic–polymer hybrid nanoparticles system with HA and polyvinyl alcohol (PVA) can, as well as proteins, deliver drugs in cancer treatment. HA has great biocompability and bone-inducing characteristics which, when connected with specific drugs like methotrexate (MTX), can be helpful in fighting off tumors [124]. Low-dimensional nanomaterials (LDNs) have a large surface area, rich binding sites, and good cellular penetration. These properties allow LDNs to be perfect drugs for cancer treatment. HA nanoparticles are selective for some tumor cells; the main mechanisms for HA are inhibition of protein synthesis, for example in mitochondria, which deregulate apoptosis. Se-doped hybrid HA can suppress the proliferation of osteosarcoma due to its low toxicity and capability to synergy with other drugs like TiO_2_/Zn co-doped HA. Together they can give promising effects in cancer treatment [125].

### 4.6. Nanohydroxyapatite in Reducing Dentin Hypersensitivity

Another important use of nanohydroxyapatite is a treatment indicated for dentin hypersensitivity. Dentin hypersensitivity has been defined as a “short, sharp pain arising from exposed dentin in response to stimuli typically thermal, evaporative, tactile, osmotic or chemical and which cannot be ascribed to any other form of dental defect or pathology” [126]. This painful clinical condition may have a negative effect on the individual’s oral health-related quality of life [127]. Statistically, about 10–30% of patients suffer from that condition [128]. The reported prevalence of dentin hypersensitivity is higher in females than in males. Although it occurs among patients of all ages, it is most often reported in the age group of 20 to 50 years, with peak prevalence from 30 to 40 years. It usually affects the canines and premolars in both the maxilla and mandible. The most affected tooth region is the cervical area of the buccal surface [129].

The reason for that kind of pain is a dentin exposure, mainly resulting from gingival recession or continuous loss of dental structure promoted by erosion, abrasion, and/or abfraction. Enamel loss can be a result of overconsumption of acidic food and tooth wear caused by stress and parafunctions. Gingival recession can be induced by an aggressive and improper technic of tooth brushing, as well as periodontal diseases. Dentin hypersensitivity is also very common after external tooth bleaching [130].

There are several theories that explain a biological mechanism of hypersensitivity. According to the “direct innervation theory” nerve endings extend through the pulp and dentin up to the dentino-enamel junction and induce impulses when they are injured, which is recognized as a characteristic for hypersensitivity to pain. The odontoblast receptor theory” finds odontoblasts as receptors which relay signal to a nerve terminal and then to the central nervous system. These impulses are relayed as pain. Both of them are currently recognized as not acceptable enough [130,131].

The hydrodynamic theory has been recently considered as the most widely accepted. Brännström and his co-workers published over 20 years’ worth of studies on both human and animal models supporting this theory. According to them, when external stimuli occurs, the fluid in the small tubules of the dentine can be rapidly moved. This is the reason for nerve terminals’ activation in the interface of the pulp and dentine, and the cause of pain [127]. This phenomenon takes place when the dentinal tubules are open. It is proven that patients suffering from dentin hypersensitivity have wider and more numerous tubules, unlike the others whose non-sensitive surfaces are covered by a smear layer [132].

According to the mechanism of dentin hypersensitivity, most treatment options focus on controlling dentin fluid movement. Thus, one of the strategies is based on therapeutic agents that promote the occlusion of the dentin tubule apertures. Nanohydroxyapatite was recently found as one of the potential elements that could be used to treat dental hypersensitivity. It is considered as one of the most biocompatible and bioactive materials, which is why it is widely used in medicine and dentistry. Nano-sized particles (20 nm) have similar morphology, size, and crystal structure compared with dental apatite [128,133]. Many reports have shown that nanohydroxyapatite has the ability to remineralize caries when it is used as a toothpaste or in topical cream form. It can easily penetrate the open dentinal tubules and strongly adsorbs to dentine apatite. As a result, the dentinal tubules become sealed. It is also responsible for maintaining a state of supersaturation of calcium, phosphate, and fluoride on the tooth surface which can be converted into the form of hydroxyapatite, fluorapatite, and calcium fluoride. The acid resistance of these compounds is similar to that of the natural tooth [128,129,134].

During last few years, many scientists decided to examine the effectiveness and safety of nanohydroxyapatite in reducing dentin hypersensitivity. The results are very promising. Vano et al. decided to compare a toothpaste containing nanohydroxyapatite with commercially available gel fluoride and placebo toothpaste. For this purpose, they created randomized double-blind clinical trial.

The results of these clinical trials show a significant reduction in dentin hypersensitivity in airblast, tactile test, and subjective evaluations. According to this, it can be concluded that nanohydroxyapatite could determine a progressive closure of the tubular openings of the dentin and cause remineralization comparable or even higher than fluoride toothpaste, to which it can be an effective alternative [135].

What is more, other scientists came to very similar conclusions. For example, Wang et al. compared the effectiveness of nanohydroxyapatite pastes indicated for professional (Desensibilize Nano-P) with or without experimental home-care application to Pro-Argin and fluoride varnish on dentin hypersensitivity relief after one and three months of treatment. They used the visual analog scale (VAS) to assess the tooth sensitivity response after standardized evaporative stimulus at baseline, and after one month and three months. Their study showed that all treatments were clinically effective, but both the Desensibilize Nano-P and Pro-Argin treatments were effective in producing relevant clinical relief for three months. The conclusion is that the agents containing nanohydroxyapatite can be as effective as other already known substances [133]. Also, the examination of Amin et al., who measured (with numeric rating scale) the level of pain related to the stimuli at the the baseline after the application of gel with nanohydroxyapatite at the intervals of 24 h, 1 month, 3 months, and 6 months, demonstrated similar results. All the 30 subjects taking part in this trial admitted to feeling significant relief as a result of treatment [130]. Figure 4 demonstrates the utilization of toothpastes and mouthwash with nanohydroxyapatite in the management of dentin and root cement hypersensitivity [136].

Nithin, John, Nagappan, Prabhu, and Senthilkumar compared the effectiveness of nanohydroxyapatite to NovaMin (bioglass). Same as others, they divided 36 subjects into 2 groups and recorded their response to various stimuli using VAS at baseline and after 4 weeks. A significant reduction in hypersensitivity was noticed in both groups, which means that nanohydroxyapatite as a new product is as effective as already tested bioglass [132].

Moreover, using nanohydroxyapatite is not only effective but also safe in everyday dental care. JM. Ramis et al. proved it with their study in 2018. They used commercially available human gingival epithelium as a non-animal alternative. It was treated with 3.1% nanohydroxyapatite for 10 min, 1 h, and 3 h. At any time points, the scientists measured MTT cell viability, LDH activity (index of cells’ death), and IL-1alpha production. Also, they used transmission electron microscopy (TEM) analysis to examine the absorption of nanohydroxyapatite in the gingival tissue. Moreover, they tested dissolution behavior of this compound in simulated gastric fluid. As a result of this study, no harmful effects were observed for human gingival epithelium tissues incubated with 3.1% nanohydroxyapatite for all-time points and parameters measured. What is more, the tested compound completely dissolved in simulated gastric fluid after 7.5 min at 37 °C. The conclusion is that oral care products containing nanohydroxyapatite are biocompatible and safe for customers [137].

Nanohydroxyapatite is a relatively new agent, the uses and pros of which are still being discovered. Recent research has shown that it can be used as an alternative method to treat dentin hypersensitivity. Several studies, the aim of which was testing the effectiveness of this material, have already been carried out, and the results are unquestionable. Moreover, studies carried out in order to prove the safety of nanohydroxyapatite let us know that there was no harmful effect which showed up during experiments. It can be concluded that nanohydroyaxpatite is a safe, biocompatible, and effective agent in reducing dentin hypersensitivity.

### 4.7. Nanohydroxyapatite in the Aspect of Bone Regeneration

Regenerative dentistry is another medical field, where nanohydroxyapatite (nano-Ha) can be successfully used considering its unique properties. In addition to improving osteointegration in dental implant surface, as described above, ceramic nanoparticles are a powerful representation of bone grafting materials [69].

The most common cause of a bone cavity is permanent tooth extraction left unreplaced. This becomes even more serious, especially when concerning multiple teeth. There are quite a lot of situations where permanent teeth must be extracted, such as irreparable tooth damage due to severe decay (37%); periodontal disease because of deep pocketing and loss of periodontal support resulting in drifting, mobility, and pain (29%); or preprosthetic extraction. However, tooth extraction is also frequently performed in orthodontics to prevent or correct malocclusion [138].

It is well established that the consequences of this uncomplicated operation might be damaging, not only because of gingival cleft or gingival recession, but also because of osseous complications in the area of procedure. Tooth extraction is followed by tissue remodeling—a reduction in the buccolingual as well as apicoronal dimension of the alveolar ridge (which means the loss of both horizontal and vertical bone height at the edentulous site) [139].

The jawbone is preserved through the pressure and stimulus of chewing. When that essential function is removed through tooth loss, the bone reabsorbs into the body. In the first year after tooth extraction 25% of the bone is lost, and this bone loss continues.

Furthermore, in addition to tooth extractions left unreplaced, bone shortage can be also caused by tumor resection, trauma, infection, and congenital anomalies. In case of a missing tooth, a dental implant can only be placed if there is sufficient bone to stabilize it properly, and for this reason it is necessary to benefit from oral surgery achievement, which is bone augmentation. It may be necessary to perform bone grafting prior to implant placement, at the time of implant placement, or subsequent to it. The mentioned procedure may be a great foundation for implant treatment, which would otherwise not be an option. Clinically, this operation takes place with the use of natural grafting materials, such as autogenous bone, allografts, and xenografts but also synthetic grafting materials [140]. “Bone graft” was defined by Bauer and Muschler as “any implanted material that alone or in combination with other materials promotes a bone healing response by providing osteogenic, osteoinductive or osteoconductive properties” [141].

Bone grafting materials can be divided into four groups on account of the way of harvesting them, mechanism of action, and maintenance inside of the bone. These four classes are as follows: autografts—patient’s own bone taken from an adjacent or remote site in the operated one. Because of providing good scaffolding for osteoconduction and containing growth factors for osteoinduction, nowadays it is still considered as a golden standard.

An allograft is a graft of tissue received from other individuals of the same species with a different genotype, whereas xenograft is taken from a donor of one species and grafted into a recipient of another species. Lastly, alloplastic grafts use synthetic foreign bodies to induce bone growth.

Autografts are systematized as bone materials; however, allografts, xenografts, and alloplastic grafts are placed in the group of osseosubstitute materials [140]. Aiming to repair the composition and function of missing tissues, today’s oral and maxillo-facial surgery benefits from tissue engineering. For clinical needs, Buser and partners have introduced the term of guided bone regeneration (GBR). GBR is a dental surgical procedure that uses barrier membranes to direct the growth of new bone for proper function, aesthetics, or prosthetic restoration.

According to M. Pietruska et al., barrier membranes are mechanical barriers that restrict the migration of epithelium and gingival connective tissue into the osseous defect. This enables bone tissue and periodontal ligament cells to recover [142]. Presently, guided bone regeneration is mainly performed in the bone cavities to support new hard tissue growth on an alveolar ridge to allow stable placement of dental implants [143]. Autologous bone graft is predominantly used in bone augmentation because of the fact that only autogenous graft has osteogenic, osteoinductive, and osteoconductive properties; however, it needs to be emphasized that autograft requires additional procedure to obtain bone from the donor site. These additional actions increase operation time and may lead to various complications, such as serious bleeding, high risk donor morbidity, and evoking pain, but also injuring the nervous or vascular tissue. Furthermore, there are limitations in available bone quantity and, equally importantly, the cost of the procedure increases, because of the need to perform additional operations [144].

As a result, our attention should be focused on bone substitute materials which are useful alternatives to autografts. Regardless of the fact that xenografts or allografts do not require multiple procedures on the same patient, they risk disease transmission and can develop immune-related complications. Taking all these problems into account, there is an increasing necessity to draw much more attention to alloplastic grafting materials [145]. The object of this review is to compare the chemical and physical properties of the two osseo substitutes mentioned below: the xenograft—Geistlich Bio-Oss^®^ and the synthetic graft—nanohydroxyapatite (nano-HA).

Bio-Oss^®^ Bone Substitute (Ed. Geistlich Soehne, Wolhusen, Switzerland) is a deproteinized, cancellous bone substitute material of bovine origin, used to fill bone defects or to reconstruct ridge configurations. This xenograft is going through a heat treatment and chemical extraction process for the purpose of complete removal of the organic components and maintaining the natural bone architecture. It acts as a scaffold onto which osteoblasts and blood vessels attach for new bone formation. Before application, Geistlich Bio-Oss^®^ is mixed with autogenous blood or a saline solution and then becomes an integral part of the newly formed bone framework and preserves volume over the long term [146]. Nanohydroxyapatite, alloplast, is the most studied biomaterial in the medical field for its proven biocompatibility and for being the main component of the non-organic part of bones and teeth. Because of its remarkable properties, such as the ability to chemically bond to bone, to not induce toxicity or inflammation, and to stimulate bone growth through a direct action on osteoblasts, nano-HA has been widely used in periodontology and in oral and maxillofacial surgery [69].

Kubasiewicz-Ross et al. performed a study on rats’ calvarial defects implanted with the new nanohydroxyapatite or well-known xenograft-Geistlich Bio-Oss^®^ to assess their osteoconductive potential. The authors pointed out that the tested material is similarly effective to the commonly used xenograft.

Figure 5 shows the application of a composite sintered scaffold with nanohydroxyapatite enriched with bovine serum albumins in socket after extraction. This new material is showing great potential in regenerative medicine [147].

As compared to the xenogenic grafting material, nHa presents several advantages. First of all, there is no risk of disease transmission, because the mentioned alloplast is a synthetic biomaterial. However, Bio-oss can induce inflammation and evoke an immunologic reaction. Additionally, there is no need to sacrifice living organisms for nano-Ha production, so gaining huge quantities of synthetic graft is eco-friendly. Moreover, there are many various methods of nanohydroxyapatite production, consequently the most economical one can be chosen. One production method in particular is considered—the precipitation process is reported to be favorable due to the ease in synthesis, cost effectiveness, and being ecological [148]. On the other hand, no statistically significant differences were found between bone regeneration with the use of the new nano-HA alloplastic material or a commercially available xenograft Bio-oss with highly proven osteoconductive potential.

Taking everything into account, nanohydroxyapatite may be the significant non-biologic graft alternative for Bio-Oss in the field of bone regeneration, including augmentation of the alveolar ridge and maxillary sinus lift augmentation [140].

## 5. Future Perspectives

Nanohydroxyapatite is undoubtedly a material commonly used in medicine due to its properties. Due to the occurrence of hydroxyapatite in bones and teeth, it has excellent biocompatibility, which gives it versatility and high potential in various branches of medicine. It is worth noting that nanohydroxyapatite allows it to be combined with various substances to further enhance its properties, e.g., antimicrobial. In the case of treatments aimed at tissue regeneration, reducing the likelihood of infection may prove crucial to the correct course of treatment [134,135,149]. Also, in the case of dental materials, nanohydroxyapatite is used to increase its mechanical and antimicrobial properties. The results of these studies are promising, and it is worth noting that the cytotoxicity of such materials is not significantly different from commonly used materials [150]. The structure of nanohydroxyapatite also increases roughness, which affects its ability to absorb substances from the environment. This allows for wide application by adding other substances. The surface area is also increased, which further increases the reactivity of the material. An example is covering implants with a layer of nHA from Human Dental Pulp Stem Cells (hDPSCs). Thanks to the nanometric size of HA particles, the potential of the coating is increased [149,151,152]. It is worth noting that the production of nanohydroxyapatite in the future will be financially beneficial due to the possibility of obtaining it from eggshells [153].

The size of nanohydroxyapatite particles is also important for tissue remineralization and combating dentin hypersensitivity. Studies have confirmed its effectiveness, which, especially in the case of hypersensitivity, which is a common condition in many people, may prove to be an effective tool in combating it. According to the authors of the study, nanohydroxyapatite has great potential, but its use in dentifrices is limited [154,155,156].

The undoubted advantage of nanohydroxyapatite is the possibility of combining it with other substances to increase specific properties. Of course, the addition of particles positively affects the properties. Therefore, the cytotoxicity of the material and its physicochemical properties should be carefully examined. The addition of, e.g., silver nanoparticles, may increase the bactericidal ability, but may also increase cytotoxicity. For this reason, it is important to test the material in vitro [157].

Due to its biocompatibility, hydroxyapatite is perfect as a drug carrier. One of the greatest challenges of modern medicine is finding drugs for various cancers. In the case of bone cancer, nanohydroxyapatite has a bright future. It can be used to transport various particles including drugs, proteins, radionuclides, and genetic material. Nanohydroxyapatite definitely has great potential, and further research is needed to better understand its capabilities in many areas of medicine. It is worth emphasizing that it is used both for basic problems such as dentin hypersensitivity and for anticancer therapy [158,159].

An interesting observation was made by Baboucarr Lowe et al.; in developed countries, due to an aging population or greater obesity, there will be more cases of osteoporosis or fractures. The properties of nanohydroxyapatite, such as biocompatibility and osteoconductivity, provide high potential in bone regeneration [160]. These properties can be further enhanced by the addition of other substances such as graphene oxide, poly(L-lactide), which provides a better scaffold structure, so there is better osteointegration [161,162].

Nanotechnology is already widely used in medicine, but it should be noted that the cytotoxicity of nanoparticles and its effect on the human body is not yet fully understood and further research is needed [163]. The problem with nanoscale drug carriers is the low loading capability; in both articles it amounted to 2%, and the different release time depending on the substance carried; a longer and more uniform time would be optimal [164,165]. In the study performed by Sobierajska P. et al., Imatinib was released much faster than Toceranib, which, in contrast, was released over a longer period of time. It should be noted that nanodrug carriers ensure accurate delivery and reduce drug resistance [165,166]. Nanohydroxyapatite is used in many anticancer therapies, not just those related to the skeletal system. Due to its high bioactivity and biocompatibility, it is possible to use it as a drug carrier in the treatment of breast, prostate or gastrointestinal cancers, among others. In the case of nHA, its cytotoxicity is not fully understood, with some studies indicating that it is tissue safe [167,168]. However, it is important to note the widespread use of nanoparticles and their combination with various substances can also have negative effects on tissues [161].

## 6. Conclusions

Nanohydroxyapatite is a material with broad potential for applications in modern medicine. The unquestionable advantage of this material is its excellent biocompatibility, which enables application in many fields of medicine. In dentistry, nanohydroxyapatite, due to its size, has very good results in combating hypersensitivity and in remineralization. It can also be used as a coating for various implants due to its osteoinductive and osteoconductive properties. The possibility of adding various substances such as metal nanoparticles can further enhance some of the abilities of nanohydroxyapatite. Nanohydroxyapatite is also being used as an anticancer drug delivery system. This is a thriving branch of medicine that can provide effective means to combat diseases that were previously difficult to treat. Nanohydroxyapatite is a substance with broad potential, so further research is needed to better understand its potential.

## Figures and Tables

**Table 1:** Examples of medical applications of the addition of metal nanoparticles to nanohydroxyapatite.

Modification of Nanohydroxyapatite	Doping Method	Possible Application	References
Addition of copper	Mechanical	Dentistry (materials)	[106]
Addition of lanthanum	Mechanical	Anticancer therapy, treatment of osteoporosis	[85,86,107]
Addition of strontium	Mechanical	Treatment of osteoporosis and osteosarcoma	[98]
Addition of other REM	Mechanical, aqueous colloidal precipitation	Treatment of osteoporosis, targeted therapy	[93,94]
Addition of magnetic dopants	Mechanical, sol–gel, aqueous colloidal precipitation	Targeted drug delivery, detoxification of biological fluids, magnetic resonance imaging (MRI), cancer therapy, magnetic fluid hyperthermia	[100]
